# Community intervention improves knowledge of HIV status of adolescents in Zambia: findings from HPTN 071-PopART for youth study

**DOI:** 10.1097/QAD.0000000000001530

**Published:** 2017-07-01

**Authors:** Kwame Shanaube, Ab Schaap, Mwate Joseph Chaila, Sian Floyd, Constance Mackworth-Young, Graeme Hoddinott, Richard Hayes, Sarah Fidler, Helen Ayles

**Affiliations:** a Zambart, Lusaka, Zambia; b Department of Paediatrics and Child Health, Desmond Tutu TB Centre, University of Stellenbosch, Cape Town, South Africa; c London School of Hygiene and Tropical Medicine; d Imperial College, London, UK.

**Keywords:** adolescents, community, HIV testing, home-based HIV counselling and testing, zambia

## Abstract

**Objective:**

To determine the uptake of home-based HIV counselling and testing (HCT) in four communities of the HPTN 071 (PopART) trial in Zambia among adolescents aged 15–19 years and explore factors associated with HCT uptake.

**Design:**

The PopART for youth study is a three-arm community-randomized trial in 12 communities in Zambia and nine communities in South Africa which aims to evaluate the acceptability and uptake of a HIV prevention package, including universal HIV testing and treatment, among young people. The study is nested within the HPTN 071 (PopART) trial.

**Methods:**

Using a door-to-door approach that includes systematically revisiting households, all adolescents enumerated were offered participation in the intervention and verbal consent was obtained. Data were analysed from October 2015 to September 2016.

**Results:**

Among 15 456 enumerated adolescents, 11 175 (72.3%) accepted the intervention. HCT uptake was 80.6% (8707/10 809) and was similar by sex. Adolescents that knew their HIV-positive status increased almost three-fold, from 75 to 210. Following visits from community HIV care providers, knowledge of HIV status increased from 27.6% (3007/10 884) to 88.5% (9636/10 884). HCT uptake was associated with community, age, duration since previous HIV test; other household members accepting HCT, having an HIV-positive household member, circumcision, and being symptomatic for STIs.

**Conclusion:**

Through a home-based approach of offering a combination HIV prevention package, the proportion of adolescents who knew their HIV status increased from ∼28 to 89% among those that accepted the intervention. Delivering a community-level door-to-door combination, HIV prevention package is acceptable to many adolescents and can be effective if done in combination with targeted testing.

## Introduction

AIDS is the leading cause of death among adolescents in Sub-Sahara Africa (SSA), and the second most common cause of death among adolescents globally [1]. An estimated 2.1 million adolescents aged 10–19 years are living with HIV globally [2], the majority of these in SSA, including 79 000 in Zambia [2–4]. More than 250 000 adolescents aged 15–19 year old were newly infected with HIV in 2013, with girls accounting for two out of three of these new infections globally and eight out of 10 in SSA [1]. The vast majority of adolescents in Africa, including those already infected with HIV, do not know their HIV status [5,6]. In SSA, only 13% of adolescent girls and 9% of adolescent boys aged 15–19 have tested for HIV and received their results in the previous 12 months [7]. Without current knowledge of HIV status, the subsequent steps in the HIV treatment cascade cannot be accessed [2,8]. Although antiretroviral therapy (ART) has dramatically improved survival for people living with HIV, clinical outcomes of HIV-positive adolescents fall behind adults, a consequence of poor knowledge of status and lack of access to therapy [4].

Uptake of HIV counselling and testing (HCT) among adolescents in Zambia remains low [9]. According to the latest Zambia Demographic Health Survey (2013–2014) report, sexually active female adolescents were more likely to have tested for HIV in the previous 12 months compared with their males counterparts (49.7 versus 26.9%) [9]. It is often more difficult for young people to seek HCT compared with adults, partly limited by the need in Zambia for all adolescents under the age of 16 years to gain parental or guardian consent prior to testing [10]. Additional barriers include fear of a positive test, stigma, association of HCT with high-risk behaviour, lack of information, perceived risk with respect to sexual exposure, poor attitudes of healthcare providers, and difficulty accessing testing services [4,9,11–18]. Such challenges result in underutilization of HCT services which subsequently leads to late diagnosis, delayed initiation of ART, poorer health outcomes, and increased transmission [4,19].

Offering home-based HCT (HB-HCT) is an important strategy for increasing HCT coverage particularly among individuals who do not use health services regularly such as adolescents [4]. Although community-based biomedical prevention approaches that include HCT have proven effective for the general population, these have remained as pilot projects for adolescents with limited evidence in SSA [20]. Robust implementation research studies are needed to provide new knowledge to improve programming and policy for highly accessible HIV testing and care, such as community-based interventions to increase adolescent HCT [21].

The HPTN 071 trial, also known as PopART (Population Effects of Antiretroviral Therapy to reduce HIV Transmission) is a three-arm community randomized trial in 12 communities in Zambia and nine communities in South Africa evaluating the impact of a combination HIV prevention package, including universal HIV testing and treatment (UTT), on community-level HIV incidence [12]. Within this study is an adolescent substudy called PopART for youth (P-ART-Y) which aims to determine the impact of the community-level HIV combination prevention packages on HIV prevalence over 26 months in adolescents aged 15–19 years. It will also assess the need for specific youth-targeted interventions in the context of community-wide UTT.

We report results and explore factors associated with HCT uptake by adolescents aged 15–19 years in four arm A communities in Zambia who are offered a ‘full’ combination HIV prevention package that includes UTT through a door-to-door approach from the P-ART-Y study. These are initial results from arm A communities only after the first 11 months of study commencement. We acknowledge that this is early data. However, there is a strong case to be made to share lessons in real time to inform policymakers and funders, hence supporting timely reporting of outcomes relevant to national and global delivery of testing and care.

## Methods

### Trial design and setting

HPTN 071 (PopART): The main trial is a cluster randomised trial being implemented in 21 high HIV prevalent communities in Zambia and South Africa [22]. The 21 communities were divided into seven triplets. Each triplet was defined as a set of three communities with similarities in geography, size, and estimated baseline HIV prevalence. Each community in a triplet was then randomly assigned to one of three arms; arm A receiving the full PopART intervention including HCT and universal ART for all HIV positives, arm B receiving the full PopART intervention with ART provided according to national guidelines, and arm C being the control arm. Further details of the trial are described elsewhere [22].

The P-ART-Y study is nested within the main HPTN 071 (PopART) trial; it is being implemented in 12 communities in Zambia and nine communities in the Western Cape Province of South Africa [22,23]. A community is defined as the catchment population of a local health unit (through which the intervention is delivered), including all schools in the selected area. The P-ART-Y study has three phases: qualitative baseline studies and collection of process data from the ongoing HPTN 071 (PopART) trial (phase 1); addition of youth-targeted interventions in communities (phase 2); and an epidemiological survey to determine the effect of the intervention on the knowledge of HIV status (phase 3).

We report on the adolescent population aged 15–19 years from the four arm A communities in Zambia using phase 1 data.

### The study intervention

The PopART combination HIV prevention package is delivered by trained community health workers called community HIV care providers (CHiPs) via a door-to-door approach, with treatment and care related services provided by local government clinics [22]. The intervention is offered in the four arm A communities and is delivered in annual rounds. CHiPs teams enumerate (list) all household members in the community including those absent, irrespective of age. They offer HCT, support for linkage to care of all identified HIV-positive individuals, ART adherence support, referral of HIV-negative males for voluntary medical male circumcision (VMMC), as well as tuberculosis (TB) and Sexually transmitted infections (STI) screening. CHiPs work in pairs within an allocated zone (consisting of 450–500 households) of a community and throughout the period, arrange repeat household visits to monitor linkage to services and offer HCT for those absent at previous visits.

CHiPs are selected by a formal process from a cadre of existing community health workers/volunteers which involves the communities. They are selected based on availability, residence in community, gender balance (58% are women), and age (median age 37 years). They receive continuous training by experienced facilitators in counselling, participatory facilitation, mentoring and follow-up, and are supported by supervisory staff who routinely visit households to check their performance.

### Informed consent

To participate in the PopART intervention, all household members aged 18 year and older are asked for verbal informed consent, whereas those younger than 18 are asked for their assent and their parents’ consent. Consent to participate does not necessarily include consent to HCT, although this is encouraged. For HCT, according to national guidelines, those 16 years and older are asked for written consent, and those under 16 years for their parent's written consent.

Individual written consent for other interventions such as VMMC is obtained using standard procedures as these are considered part of the routine delivery of HIV prevention services and not specifically study related.

### HIV counselling and testing

HCT is offered to all household members, those who agree have the option to receive HCT as couples, a household group or individually. HIV testing is conducted according to the Zambian national algorithm that uses Alere Determine HIV-1/2 test (Alere International Limited, Japan) as a screening test and the Unigold HIV test (Trinity Biotech Manufacturing Ltd, Bray, Ireland) as the confirmatory test for individuals who have a reactive result on the screening test. Individuals with discordant screening and confirmatory tests have repeat tests immediately and repeated after 2 weeks if results are still discordant.

### Data collection and analysis

In the main trial, the intervention has been offered to all household members 18 years and older since December 2013. Data is recorded in an electronic data capture device. Collection of additional data on the uptake of the intervention for those under 18 years began in October 2015 following ethical approvals. We report on data collected from October 2015 to September 2016 for adolescents aged 15–19 years from four arm A communities in Zambia. Arm A communities only were included in this analysis as they all received the same combination prevention package and no comparisons with standard of care communities was done.

Univariable and multivariable random-effects logistic regression was used to estimate unadjusted odds ratio (OR) and adjusted (aOR), with all analysis controlled for community. Analysis was done separately for males and females, with zone as a random effect to account for clustering because of variation in CHiPs’ performance. Association between HCT uptake and variables that include age, sex, community, time since previous HIV test and presence of young or older adults in the household was explored. The likelihood ratio test was used to compare the fit of different models and quantify evidence of associations between individual characteristics and HCT uptake. In multivariable models, age group and prior history of HCT were considered for inclusion first, and confounding among community, age group, and previous history of HCT was explored. Following this, other factors were considered and those found to be association with the outcome (*P* < 0.10) in multivariable analysis were included. Analysis was restricted to individuals with complete data.

Knowledge of HIV status before the intervention was defined as self-reported HIV positive or self-report to have tested HIV negative in the previous 12 months. Knowledge of HIV status after intervention was defined as self-reported HIV positive, tested by CHiPs or tested HIV negative elsewhere in the previous 12 months. Comparison of knowledge of HIV status by age and sex was done as these are widely acknowledged risk factors [24].

To calculate HIV-prevalence, we assumed that all adolescents who knew their HIV-positive status self-reported this to the CHiPs and that the proportion of HIV positives in those not accepting testing was the same as those accepting testing. HIV prevalence was defined as the sum of self-reported HIV positive, tested positive by CHiPs and those estimated to be HIV positive among those declining testing, divided by the number of adolescents consenting.

To calculate the United Nations Programme on HIV/AIDS (UNAIDS) ‘first 90’ among consenters and the population we assumed that all consenting adolescents who knew HIV-positive status, self-reported this to the CHiPs; the proportion of HIV positives who knew their status was similar in those not consenting as in those consenting (at time of CHiPs visit); the proportion of those testing HIV positive among those accepting testing by CHiPs was the same in those consenting to the intervention but declining testing and those estimated to be eligible for HIV testing in the group not consenting to the intervention. The ‘first 90 before the intervention’ defines those who knew their HIV-positive status among those estimated to be HIV positive before the CHiPs’ visit. The ‘first 90 after the intervention’ defines the same but immediately after the CHiPs’ visit and includes the impact of HCT.

### Ethical approval

Ethics approval was obtained from the ethics committees of the University of Zambia and the London School of Hygiene and Tropical Medicine. Permission to conduct the study was received from the Ministry of Health.

## Results

### Participation

**Fig. 1:**
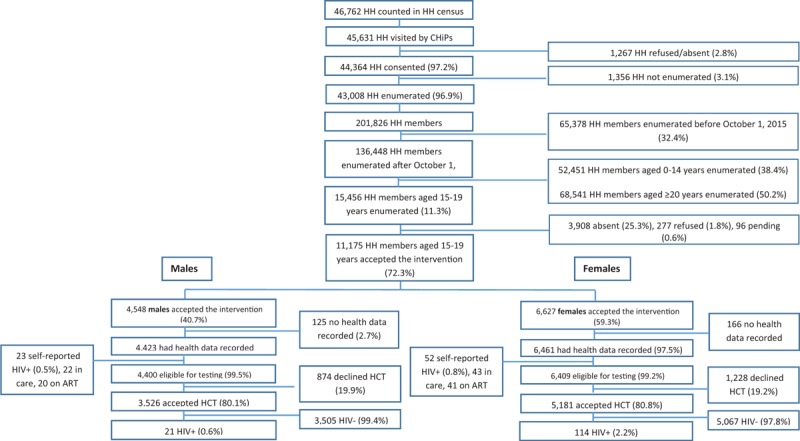
Flow chart showing study intervention participation and HIV-testing eligibility.

ART, antiretroviral therapy; CHiPs, community HIV care providers; HCT, HIV counselling and testing; HH, household.

From June 2015 to September 2016, 97.6% (45 631/46 762) of the total households were visited by CHiPs (Fig. 1). Enumeration of households was completed for 94.2% (43 008/45 631) and average household size was 4.7 individuals (201 826 members in 43 008 households).

In total, 15 456 adolescents aged 15–19 were enumerated and 277 (1.8%) refused to participate in the intervention. Refusal was highest among the 18-year olds (95/3,450; 2.8%). Refusal was higher in males than females (150/6957; 2.2% versus 127/8499; 1.5%). CHiPs were not able to contact 3,908/15,456 (25.3%) adolescents. For males 31.9% (2,219/6,957) could not be contacted and for females 19.9% (1689/8499). 15-year olds adolescents were more difficult to contact (1036/2786; 37.2%) than older ages.

A total of 72.3% (11 175/15 456) adolescents accepted the intervention (40.7% (4548/11 175) males; 59.3% (6627/11 175) females). For 10 884/11 175 (97.4%) adolescents, health data was electronically recorded after accepting the intervention. In total 75/10 884 (0.7%) adolescents self-reported to be HIV positive; leaving 10 809 adolescents eligible for HIV testing (Fig. 1). Of the self-reported HIV positives, more males than females were linked to care (22/23 males, 95.6%; 43/52 females, 82.7%). Most adolescents were on ART (20/22 males, 90.9%; 41/43 females, 95.3%).

### HIV counselling and testing uptake

Overall 80.6% (8707/10 809) adolescents accepted HIV testing by the CHiPs. HCT uptake was similar by sex (80.8% females; 80.1% males; Fig. 1). Main reasons for refusing testing varied per age. Among younger age groups the main reason for refusal was ‘not considered at risk’ (37% in 15-year old males; 32% in 15-year old females), whereas among older adolescents ‘recently tested’ was the main reason for refusal (35% in 19-year old males; 44% in 19-year old females). On average about 19% of decliners gave ‘other’ reasons for refusing to be tested, 15% were ‘too busy’, 11% did not give a reason and some feared the result of the test (1.5%) or the finger prick (1.5%) (Data not shown).

Over half of the adolescents had never been tested for HIV before [60.6% (2665/4400) males and 53.1% (3406/6409) females]. There was a trend between age and potential first time testers whereby younger adolescents were less likely to have tested before compared with older ones [79.8% (1304/1634) never tested in 15-year olds and 30.3% (846/2790) in 19-year olds].

**Fig. 2:**
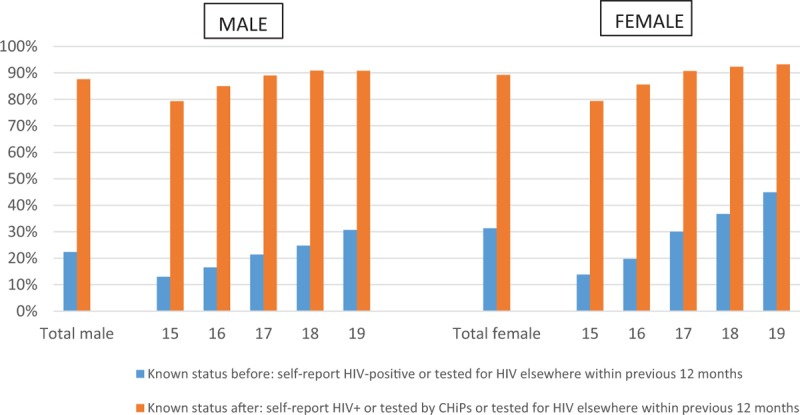
Knowledge of HIV-status before and after the intervention by age and sex.

Of consenting adolescents, 27.6% (3007/10 884) knew their HIV status before the intervention and 88.6% (9636/10 884) after the intervention. For males, the increase was from 22.3% (986/4423) to 87.6% (3876/4423); for females from 31.3% (2021/6461) to 89.1% (5760/6461; Fig. 2). The highest impact of the intervention in terms of knowing one's HIV status was among 16-year old males (16.5% before to 85.0% after the intervention) and 16-year old females (19.7% before to 85.6% after the intervention).

### Factors associated with HIV counselling and testing uptake

In univariable analysis, factors associated with HCT uptake were community, age, time since previous HIV test, having at least one adult in the household previously tested by CHiPs, and being symptomatic for STIs (Table 1). Additionally, for males, circumcision was associated with HCT uptake, whereas for females having a known HIV-positive young adult (aged 20–34) in the household was associated with HCT uptake. These associations were maintained in the multivariable analysis, with the exception of STI symptoms among males.

**Table 1:** Factors associated with uptake of home-based HIV counselling and testing by sex.

	Males			Univariate			Multivariate			Females			Univariate			Multivariate		
Characteristic	*N*	Accept HIV test	%	OR	95% CI	*P*	OR	95% CI	*P*	*N*	Accept HIV test	%	OR	95% CI	*P*	OR	95% CI	*P*
Total	4400	3526	80.1							6409	5181	80.8						
Community						<0.001			<0.001						<0.001			<0.001
Z2	583	471	80.8	1.15	0.65–2.02		0.87	0.49–1.57		850	688	80.9	1.06	0.63–1.78		0.92	0.54–1.58	
Z5	1207	1097	90.9	3.74	2.34–6.01		2.90	1.77–4.75		1717	1559	90.8	3.19	2.08–2.89		2.80	1.81–4.34	
Z8	1982	1506	76.0	1.00			1.00			2899	2239	77.2	1.00			1.00		
Z10	628	452	72.0	0.77	0.49–1.21		0.73	0.45–1.18		943	695	73.7	0.75	0.49–1.14		0.87	0.57–1.35	
Age						<0.001			<0.001						<0.001			<0.001
15	681	504	74.0	1.00			1.00			944	696	73.7	1.00			1.00		
16	829	643	77.6	1.27	0.98–1.64		1.42	1.08–1.87		1197	962	80.4	1.52	1.23–1.89		1.69	1.35–2.13	
17	745	610	81.9	1.74	1.33–2.29		2.03	1.51–2.72		997	816	81.8	1.80	1.43–2.27		2.27	1.77–2.90	
18	1049	867	82.7	1.73	1.35–2.23		2.27	1.72–3.00		1610	1346	83.6	1.87	1.52–2.30		2.53	2.02–3.18	
19	1096	902	82.3	1.68	1.31–2.16		2.22	1.68–2.94		1661	1361	81.9	1.63	1.33–2.00		2.41	1.91–3.05	
Previous HIV test						<0.001			<0.001						<0.001			<0.001
Not tested	2665	2235	83.9	1.00			1.00			3406	2846	83.6	1.00			1.00		
0–3 months (recently tested)	326	135	41.4	0.15	0.11–0.19		0.11	0.08–0.15		746	404	54.2	0.24	0.20–0.30		0.20	0.17–0.25	
4–6 months	278	209	75.2	0.63	0.46–0.87		0.52	0.37–0.72		513	431	84.0	1.12	0.86–1.47		0.90	0.68–1.19	
7–9 months	187	152	81.3	1.00	0.67–1.51		0.71	0.46–1.08		384	328	85.4	1.17	0.85–1.60		0.96	0.69–1.33	
10–12 months	178	144	80.9	0.98	0.64–1.50		0.68	0.44–1.06		340	286	84.1	1.21	0.88–1.67		0.97	0.69–1.35	
More than12 months	735	627	85.3	1.15	0.60–1.46		0.90	0.69–1.18		1001	869	86.8	1.30	1.05–1.62		1.02	0.81–1.29	
Unknown	31	24	77.4	0.58	0.23–1.46		0.51	0.19–1.38		19	17	89.5	1.37	0.29–6.40		1.21	0.25–5.81	
Young adults 20-34 in HH ever tested by CHiPs						<0.001			<0.001						<0.001			<0.001
No young adult tested	2048	1547	75.5	1.00			1.00			3126	2393	76.6	1.00			1.00		
1 young adult tested	1470	1211	82.4	1.52	1.27–1.82		1.61	1.33–1.96		2170	1810	83.4	1.48	1.27–1.72		1.53	1.31–1.79	
2 young adults tested	562	486	86.5	1.98	1.50–2.61		1.95	1.46–2.62		751	664	88.4	2.30	1.79–2.95		2.26	1.74–2.94	
3 or more young adults tested	320	282	88.1	2.26	1.56–3.28		2.16	1.46–3.20		362	314	86.7	1.83	1.31–2.54		1.59	1.13–2.25	
Older adults = 35 in HH ever tested by CHiPs						0.002			0.002						0.001			0.001
No older adult tested	2197	1700	77.4	1.00			1.00			3727	2933	78.7	1.00			1.00		
1 older adult tested	1459	1,204	82.5	1.32	1.10–1.59		1.31	1.08–1.59		1813	1513	83.5	1.34	1.14–1.56		1.36	1.15–1.60	
2 or more older adults tested	744	622	83.6	1.37	1.08–1.74		1.43	1.10–1.84		869	735	84.6	1.39	1.12–1.73		1.42	1.13–1.78	
Known HIV-positive young adults 20–34 in HH						0.195									0.001			0.007
No	4079	3258	79.9	1.00						5911	4750	80.4	1.00			1.00		
Yes	321	268	83.5	1.24	0.89–1.72					498	431	86.5	1.56	1.18–2.06		1.50	1.11–2.03	
Known HIV-positive older adults = 35 in HH						0.712									0.064			0.004
No	3954	3154	79.8	1.00						5806	4685	80.7	1.00			1.00		
1 older adult HIV-positive	404	312	77.2	0.89	0.69–1.17					493	411	83.4	1.35	1.04–1.75		1.60	1.21–2.12	
2 or more older adults HIV-positive	81	60	74.1	0.96	0.55–1.68					110	85	77.3	0.98	0.60–1.58		1.09	0.66–1.82	
TB						0.189									0.290			
Not symptomatic	4385	3513	80.1	1.00						6379	5154	80.8	1.00					
On treatment	1	1	100.0							5	4	80.0	1.03	0.11–9.96				
TB suspects	14	12	85.7	2.67	0.54–13.09					25	23	92.0	2.83	0.65–12.43				
Pregnant															0.269			
No										6001	4853	80.9	1.00					
Yes										371	300	80.9	0.85	0.64–1.13				
Missing										37	28	75.7						
STI						0.034									<0.001			0.003
Not symptomatic	4363	3494	80.1	1.00						6329	5113	80.8	1.00			1.00		
Symptomatic	21	20	95.2	5.62	0.73–43.30					59	56	94.9	4.97	1.52–16.25		4.62	1.38–15.50	
Missing	16	12	75.0							21	12	57.1						
Circumcision						0.025			<0.001									
Not circumcised	2405	1929	80.2	1.00			1.00											
VMMC	1738	1405	80.8	1.15	0.96–1.37		1.38	1.15–1.68										
Traditional	138	105	76.1	0.64	0.41–0.99		0.68	0.42–1.10										
Missing	119	87	73.1															

Results not shown: Co-variants not associated with outcome (HB-HCT uptake) in multivariable analyses. CHiPs, community HIV care providers; CI, confidence interval; HB-HCT, home-based HIV counselling and testing; OR, odd ratio; STI, sexually transmitted infections; TB, tuberculosis; VMMC, voluntary medical male circumcision.

Older adolescents aged 17–19 had more than two-fold higher odds of accepting HCT than 15-year olds. Also 16-year olds were more likely to accept HCT than 15-year olds but the effect was less pronounced [aOR: 1.42, 95% confidence interval (CI) 1.08–1.87 males; 1.69, 95% CI 1.35–2.13 females).

There was no difference observed in HCT uptake in communities Z2, Z10, and Z8. In community Z5, adolescents had three-fold higher odds of accepting HCT, compared with community Z8 (aOR: 2.90, 95% CI: 1.77–4.75 males; 2.80, 95% CI 1.81–4.34 females). Adolescents, recently, tested for HIV had the lowest odds of HCT uptake compared with those who never tested (aOR: 0.11, 95% CI 0.08–0.15 males; 0.20, 95% CI 0.17–0.25 females).

Adolescents living in households with additional one or more young (aged 20–34 years) or older (aged ≥35 years) adult previously tested by CHiPs had higher odds of accepting HCT compared with households with no household member previously tested by CHiPs (Table 1). Additionally, in females, having an HIV-positive household member (young or older adult) in the same household was associated with higher HCT uptake (aOR: 1.60, 95% CI 1.21–2.12).

There was weak evidence that HCT uptake was associated with being symptomatic for STI (OR: 5.62, 95% CI 0.73–43.30 in males and 4.97, 95% CI 1.52–16.25 in females) and TB (OR: 2.67, 95% CI 0.54–13.09 in males and 2.83, 95% CI 0.65–12.43 in females) in univariable analysis. However, there was moderate evidence for an association for STI symptomatic females in multivariable analysis (aOR 4.62, 95% CI 1.38–15.50). VMMC was associated with slightly higher HCT uptake (aOR: 1.38, 95% CI 1.15–1.68), whereas traditional circumcision was weakly associated with lower HCT uptake (aOR: 0.68; 95%CI: 0.42–1.10), compared with no circumcision.

### Proportion of HIV positives among those who consented

#### Self-reported HIV positives

Overall 0.5% (23/4423) of males and 0.8% (52/6461) of females self-reported to be HIV positive, varying by community and age (Table 2 and Table 3). Female adolescents self-reporting to be HIV positive increased with age, whereas this was less pronounced in males. Most adolescents who self-reported to be HIV positive stated to have been last tested more than 12 months ago (23/23 males and 33/52 females).

**Table 2:** Self-reported HIV-positive and newly HIV positive identified by community HIV care providers in adolescent males.

Characteristic	Total consented to intervention	Self-reported HIV^+^	Tested HIV^+^	Known HIV^+^ after the intervention	Proportion of HIV^+^ diagnosed by CHiPs	HIV^−^ prevalence among consenters
Total	4423	23	21	44	47.7%	1.1%
Community						
Z2	588	5	4	9	44.4%	1.7%
Z5	1207	0	7	7	100.0%	0.6%
Z8	1994	12	8	20	40.0%	1.1%
Z10	634	6	2	8	25.0%	1.4%
Age						
15	685	4	6	10	60.0%	1.8%
16	831	2	1	3	33.3%	0.4%
17	751	6	3	9	33.3%	1.3%
18	1053	4	4	8	50.0%	0.8%
19	1103	7	7	14	50.0%	1.4%
Previous HIV test						
Not tested	2816	0	17	17	100.0%	0.7%
0–3 months	326	0	0	0		0.0%
4–6 months	274	0	2	2	100.0%	1.0%
7–9 months	181	0	0	0		0.0%
10–12 months	162	0	1	1	100.0%	0.8%
more than 12 months	635	23	1	24	4.2%	3.8%
Unknown	29	0	0	0		0.0%
Young adults aged 20-34 in HH ever tested by CHiPs						
No young adult tested	2062	14	9	23	39.1%	1.3%
1 young adult tested	1477	7	7	14	50.0%	1.0%
2 young adults tested	564	2	5	7	71.4%	1.4%
3 or more young adults tested	320	0	0	0		0.0%
Older adults aged 35 or older in HH ever tested by CHiPs						
No older adult tested	2212	15	13	28	46.4%	1.4%
1 older adult tested	1466	7	8	15	53.3%	1.1%
2 or more older adults tested	745	1	0	1	0.0%	0.1%
Known HIV-positive young adults aged 20-34 in HH						
No	4100	21	20	41	48.8%	1.1%
Yes	323	2	1	3	33.3%	1.0%
Known HIV-positive older adults aged 35 or older in HH						
No	3926	11	18	29	62.1%	0.9%
1 older adult HIV-positive	412	8	3	11	27.3%	2.9%
2 or more older adults HIV-positives	85	4	0	4	0.0%	4.7%
TB						
Not symptomatic	4405	20	18	38	47.4%	1.0%
On treatment	1	0	0	0		0.0%
TB suspects	17	3	3	6	50.0%	38.2%
STI						
Not symptomatic	4385	22	21	43	48.8%	1.1%
Symptomatic	22	1	0	1	0.0%	4.5%
Missing	16	0	0	0		0.0%
Circumcision						
Not circumcised	2425	20	16	36	44.4%	1.6%
VMMC	1741	3	2	5	40.0%	0.3%
Traditional	138	0	2	2	100.0%	1.9%
Missing	119	0	1	1	100.0%	1.1%

CHiPs, community HIV care providers; HH, household; HIV+, HIV positive; HIV-, HIV negative; STI, sexually transmitted infections; TB, tuberculosis; VMMC, voluntary medical male circumcision.

**Table 3:** Self-reported HIV-positive and newly HIV-positive identified by community HIV care providers in adolescent females.

Characteristic	Total consented to intervention	Self-reported HIV^+^	Tested HIV^+^	Known HIV^+^ after the intervention	Proportion of HIV^+^ diagnosed by CHiPs	HIV^−^prevalence among consenters
Total	6461	52	114	166	68.7%	3.0%
Community						
Z2	855	5	14	19	73.7%	2.6%
Z5	1729	12	29	41	70.7%	2.5%
Z8	2924	25	57	82	69.5%	3.4%
Z10	953	10	14	24	58.3%	3.0%
Age						
15	949	5	3	8	37.5%	1.0%
16	1204	7	15	22	68.2%	2.1%
17	1001	4	18	22	81.8%	2.6%
18	1620	10	43	53	81.1%	3.8%
19	1687	26	35	61	57.4%	4.1%
Previous HIV test						
Not tested	3565	4	55	59	93.2%	2.0%
0–3 months	754	8	4	12	33.3%	2.0%
4–6 months	505	1	12	13	92.3%	3.0%
7–9 months	377	5	5	10	50.0%	2.9%
10–12 months	315	1	9	10	90.0%	3.7%
more than 12 months	926	33	29	62	46.8%	7.2%
Unknown	19	0	0	0		0.0%
Missing						
Young adults aged 20-34 in HH ever tested by CHiPs						
No young adult tested	3156	30	56	86	65.1%	3.3%
1 young adult tested	2186	16	41	57	71.9%	3.0%
2 young adults tested	754	3	13	16	81.3%	2.3%
3 or more young adults tested	365	3	4	7	57.1%	2.1%
Older adults aged 35 or older in HH ever tested by CHiPs						
No older adult tested	3759	32	73	105	69.5%	3.3%
1 older adult tested	1826	13	26	39	66.7%	2.4%
2 or more older adults tested	876	7	15	22	68.2%	2.8%
Known HIV-positive young adults aged 20-34 in HH						
No	5949	38	100	138	72.5%	2.7%
Yes	512	14	14	28	50.0%	5.9%
Known HIV-positive older adults aged 35 or older in HH						
No	5846	40	99	139	71.2%	2.8%
1 older adult HIV-positive	501	8	12	20	60.0%	4.5%
2 or more older adults HIV-positive	114	4	3	7	42.9%	6.9%
TB						
Not symptomatic	6427	48	107	155	69.0%	2.8%
On treatment	5	0	0	0		0.0%
TB suspects	29	4	7	11	63.6%	40.0%
Pregnant						
No	6049	48	96	144	66.7%	2.8%
Yes	375	4	16	20	80.0%	6.3%
Missing	37	0	2	2	100.0%	7.1%
STI						
Not symptomatic	6379	50	103	153	67.3%	2.8%
Symptomatic	61	2	11	13	84.6%	22.3%
Missing	21	0	0	0		0.0%

CHiPs, community HIV care providers; HH, household; HIV+, HIV positive; HIV-, HIV negative; STI, sexually transmitted infections, TB, tuberculosis.

#### Newly diagnosed HIV positives

Of all adolescents aware of their HIV positive status after the intervention, 47.7% (21/44) of males and 68.7% (114/166) of females were diagnosed by CHiPs. The biggest attribution was made in females aged 17 and 18 years, where over 80% of the known HIV positives were diagnosed by CHiPs (Tables 2 and 3). The number of adolescents known to have HIV increased from 75 (23 males; 52 females) to 210 (44 males; 166 females) (Tables 2 and 3). The relative benefit for the intervention varied between communities.

#### HIV prevalence

Overall HIV prevalence among consenters was higher in females than males (3.0% compared with 1.1%). As expected, the highest prevalence was found in those with symptoms suggestive of TB and STI for females.

#### ‘First 90’

**Fig. 3:**
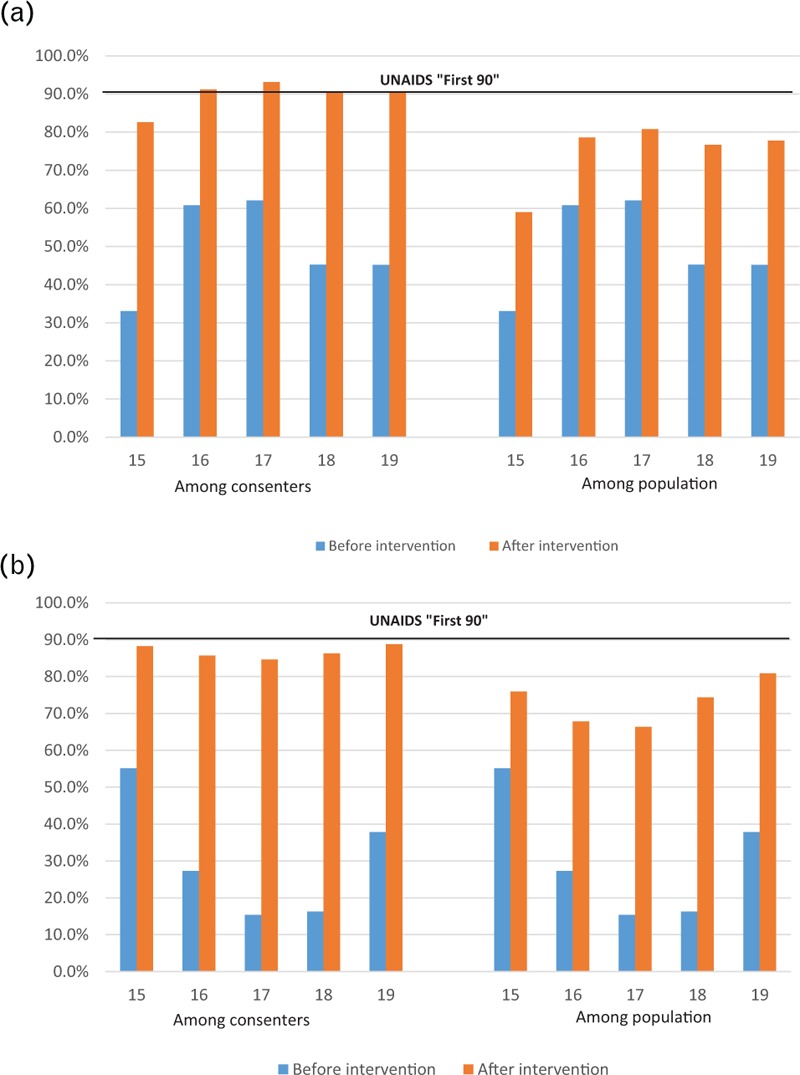
(a) Proportion of HIV-positive adolescent males that know their status “First 90” (b) Proportion of HIV-positive adolescent females that know their status “First 90”.

All HIV-positive male adolescents’ consenters reached the ‘first 90’ after the intervention except for the 15-year olds, whereas in females proportions were approaching the first 90 across all ages. The ‘first 90’ in the population was not equally reached in both sexes (Fig. 3 a and b).

## Discussion

### Uptake of home-based HIV counselling and testing

The study has shown that after almost 1 year of delivering a HB-HCT approach, the proportion of 15–19-year old adolescents who know their HIV status tripled from 27.6 to 88.6%, approaching the target goal of 90%. To our knowledge, this study is the first community-based population intervention of this size that demonstrates the feasibility of delivering HB-HCT for adolescents aged 15–19 years in SSA. We show that in a high HIV-prevalence generalized epidemic, HB-HCT is acceptable and feasible for adolescents and has made a significant impact toward the first of UNAIDS 90–90–90 targets [2].

Our findings are consistent with prior research that shows that high uptake of HCT among adolescents can be achieved through home-base interventions [24]. In Uganda, home-based family-centred HCT resulted in a six times increase of HCT uptake among individuals aged 6 to 14 years compared with that provided at clinics [24,25]. Not only do home-based approaches achieve high HCT uptake among adolescents, they also identify individuals at early stage of their HIV infection, as they do not rely on individuals presenting to health facilities with symptoms [24].

We were not able to reach 27.7% of the enumerated adolescents mostly because of absent rates especially among males and younger age groups. For such harder-to-access groups, HB-HCT can be implemented alongside other methods that have been demonstrated to increase testing rates, including provider initiated testing and counselling [26], adolescent-friendly healthcare facilities [21], social marketing campaigns [27], mobile testing [28] as well as self-testing [29]. A systematic review, investigating acceptability of HCT in individuals aged 5–19 years in SSA found that provider initiated testing and counselling achieved the highest acceptability (86%), followed by HB-HCT (84.9%) but rates were lower for school-linked HCT (60.4%) and family-centred approaches (51.7%) [24].

Although HCT uptake was similar by sex, HIV prevalence was higher in females compared with males, as observed elsewhere [10,30–32]. Adolescent girls have earlier sexual debut than boys because of factors such as intergenerational sex [31], gender-based violence, economic dependency, and low ability to negotiate condom use [33,34]. This has implications for future interventions which need to prioritize adolescent girls.

### Factors associated with uptake of home-based HIV counselling and testing

Several factors at individual, household and community levels have been associated with HCT uptake [39]. Although these are widely acknowledged in adults, evidence is limited for adolescents. We found that HCT uptake was associated with age, community, duration since previous HIV test, having young adults in the household previously tested by CHiPs, being symptomatic for STIs, VMMC and having a known HIV-positive adult in the household.

Increasing age corresponded with increasing HCT uptake partly because of 15–17 years olds being less regularly found at home compared with 18–19 years old because of school attendance [9]. This raises the potential role of testing these young adolescents through other sources. Furthermore, the refusal rate among 15 year olds was particularly high. The age of consent for HCT services in Zambia is 16 years [35]; those younger encountered more obstacles to HCT around obtaining parental consent. This supports the need to engage parents and increase their awareness of the benefits of HCT [21,36]. The younger adolescent found to be HIV positive are most likely to have been perinatally infected, and although the relative numbers are thought to be small, may represent a specific group to target for alternative routes of offering testing, for example through parents’ ART clinics.

Contextual factors have been shown to influence uptake of interventions like HCT. Detailed description of contextual differences in our communities is described elsewhere [23]. High levels of HIV sensitization programs conducted by non-governmental organizations have been observed in community Z5, potentially contributing to the high HCT uptake [23]. Community Z5 is dominated by a largely informal sector, where access to clinics and services may be limited because of poverty and testing options situated outside the community [23].

Interestingly, we show that household composition influences HCT uptake. In females, having an HIV-positive household member (young or older adult) was associated with higher HCT uptake and to our knowledge has not been demonstrated before in this age group. This finding supports the need for intensifying HIV index case testing for adolescents in households that have an HIV-positive adult. Index case testing of individuals receiving any HIV care or treatment service has been recommended, either through facilities or home based [37]. In adults, index testing has been effective through assisted partner notification services [38–40]. In children, index case testing has been shown to have a 10-fold increase in the identification and enrolment of HIV-infected children into paediatric care and treatment services [41]. However, there is little known about the feasibility of index case testing amongst adolescents. This finding emphasises the potential role index case testing could have among adolescents, and the need for further research.

In this study, the intervention increased opportunities to engage and influence other family members as adolescents living in households with at least an additional member previously tested by CHiPs had higher odds of accepting HCT; as reported elsewhere [15].

Although few adolescents reported STI symptoms, females symptomatic for STIs were more likely to test for HIV. Previous research on this is mixed: in Mwanza, Tanzania, STI symptoms did not correlate with HCT uptake [42], whereas another study in Tanzania showed an association between testing uptake and STI symptoms [43]. These mixed results may be because of the challenging nature of self-reporting of STI symptoms and the perceived increased risk of HIV acquisition. Males who had received VMMC had higher HCT uptake; it is hypothesized that individuals who are more likely to have VMMC may be more willing to accept medical interventions, including HCT.

### Limitations

In this study, ‘first 90’ was not quite reached partly because of challenges around parental consent; parents were often not found at home, or were not willing to provide consent for younger adolescents. The intervention is resource intensive and the overall cost-effectiveness will not be available until completion of the main trial. It may be more feasible to deliver streamlined and targeted youth-friendly approaches for different age bands and sex similar to index-case based testing programs [37]. Furthermore, data was only analysed from the intervention arm A communities, without comparison with the control communities, where data is not yet available. However, as the majority of adolescents tested through the intervention, we feel confident in attributing the large rise in HCT to the intervention.

## Conclusion

Through a home-based approach of offering a combination HIV prevention package the proportion of adolescents who knew their HIV status increased from ∼28 to 89%, approaching the target goal of 90%. This study provides strong evidence that HB-HCT is feasible, acceptable and effective at significantly increasing HCT uptake among adolescents aged 15–19. HB-HCT is an effective intervention to increase the uptake of the first of UNAIDS 90–90-90 targets amongst adolescents and could be particularly effective if implemented in combination with targeted testing.

## Acknowledgements

We would like to acknowedge the HPTN 071 (PopART) and P-ART-Y study teams.

The content is solely the responsibility of the authors and does not necessarily represent the official views of the NIAID, NIMH, NIDA, PEPFAR, 3ie, or the Bill & Melinda Gates Foundation. We are grateful to all members of the HPTN 071 (PopART) and P-ART-Y Study Teams, and to the study adolescents and their communities, for their contributions to the research.

K.S. took the lead on writing the paper together with M.J.C. and C.M-Y. A.S. led on the statistical analysis together with S.F. All other authors were involved in the design and implementation of the study; provided guidance and approved the final version of the paper.

HPTN 071 is sponsored by the National Institute of Allergy and Infectious Diseases (NIAID) under Cooperative Agreements UM1-AI068619, UM1-AI068617, and UM1-AI068613, with funding from the U.S. President's Emergency Plan for AIDS Relief (PEPFAR). Additional funding is provided by the International Initiative for Impact Evaluation (3ie) with support from the Bill & Melinda Gates Foundation, as well as by NIAID, the National Institute on Drug Abuse (NIDA) and the National Institute of Mental Health (NIMH), all part of the U.S. National Institutes of Health (NIH).

The P-ART-Y study is funded by Evidence for HIV Prevention in Southern Africa (EHPSA), a Department for International Development (DFID) programme managed by Mott MacDonald.

### Conflicts of interest

There are no conflicts of interest.
